# Synergistic Physical–Chemical Immobilization of Mn in Electrolytic Manganese Residue Co-Solidified with Sludge Biochar: Interfacial Speciation, Pore Evolution, and LCA

**DOI:** 10.3390/ma19214451

**Published:** 2026-10-22

**Authors:** Xuquan Huang, Xinyi Jia, Xiaorong Zhao, Shuhua Liu, Xin Chen, Chenqiong Ye, Haojie Wang, Fei Xue, Zilong Han, Yuwei Xiang

**Affiliations:** 1 Engineering Research Center of Eco-Environment in Three Gorges Reservoir Region, Ministry of Education, China Three Gorges University, Yichang 443002, China; 2 College of Hydraulic & Environmental Engineering, China Three Gorges University, Yichang 443002, China; 3 Yichang Key Laboratory of Solid Waste Disposal and Resource Utilization, China Three Gorges University, Yichang 443002, China; 4 State Key Laboratory of Water Resources Engineering and Management, Wuhan University, Wuhan 430072, China; 5 China Construction Third Engineer Bureau Group Yangtze River Co., Ltd., Wuhan 430064, China

**Keywords:** electrolytic manganese residue, sewage sludge biochar, synergistic physical-chemical immobilization, pore tortuosity, life cycle assessment

## Abstract

The long-term immobilization of highly mobile Mn^2+^ in electrolytic manganese residue (EMR) remains a critical challenge for sustainable waste management, as conventional cement-based solidification often suffers from carbon-intensive clinker demand and insufficient interfacial stabilization. Herein, we propose a co-solidification strategy integrating municipal sewage sludge-derived biochar (SBC) into cementitious matrices to enhance Mn immobilization. Multi-scale characterization revealed that SBC functioned as a micro-aggregate to increase pore network tortuosity by 124% and further modified the hydration/interfacial environment of the cementitious matrix. BCR sequential extraction showed that the acid-soluble/exchangeable Mn fraction decreased from 63% in raw EMR to 41% in ECB without SBC and further to 28% after the incorporation of 5 wt% SBC, indicating a progressive redistribution of Mn toward less mobile fractions. These coupled chemical and physical effects reduced Mn leaching from 14.5 mg/L to 0.2 mg/L after 28 days of curing, corresponding to a 98.6% reduction and meeting the relevant leaching criterion for non-hazardous classification. Mechanistic analysis indicated that Mn immobilization was governed by the combined effects of hydration-product association, interfacial interactions, matrix densification, and pore-network refinement. Life-cycle assessment further showed that the climate benefit of the SBC route was dependent on the sludge-management scenario, particularly the energy demand for sludge pretreatment and drying, auxiliary fuel use, and energy recovery. This study elucidates the interfacial engineering principles of SBC-amended cementitious systems and offers a technically feasible pathway for the co-valorization of industrial and municipal solid wastes.

## 1. Introduction

The valorization of solid wastes as functional additives provides a promising strategy for the synergistic treatment and stabilization of industrial wastes [1,2]. The massive accumulation of electrolytic manganese residue (EMR) poses a severe environmental burden because of the high mobility and leachability of residual Mn [3,4]. Conventional cement-based solidification/stabilization (S/S) has been widely investigated for EMR management, as the alkaline environment and hydration products generated in cementitious matrices can reduce Mn mobility through precipitation, physical encapsulation, and interactions with solid phases [3,5,6]. However, conventional cement-based S/S still faces limitations related to the stability of immobilized metals and the environmental impacts associated with clinker production [2,5,6]. Therefore, improving Mn immobilization while reducing reliance on conventional cementitious binders remains an important issue for the sustainable management of EMR.

Recent studies on multi-waste binders containing EMR have demonstrated that the co-utilization of different solid wastes can improve the performance of cementitious matrices [7]. Meanwhile, advances in interfacial engineering have highlighted the potential of biochar derived from waste biomass as a multifunctional additive in cementitious systems [8,9]. Beyond its well-established role in aqueous pollutant adsorption, biochar possesses a unique combination of properties, including a hierarchical porous structure, high specific surface area, and abundant surface functional groups, which can influence the retention and transport of heavy metals at solid–liquid interfaces [10,11]. When incorporated into cementitious matrices, biochar may refine the pore structure, modify the interfacial transition zone (ITZ), and influence water redistribution and subsequent hydration processes [8,9,12]. In addition to these physical and interfacial effects, sludge-derived carbonaceous materials have been reported to exhibit electron-donating and electron-mediating capacities, indicating their potential involvement in interfacial redox reactions [13].

Current strategies for EMR passivation have mainly focused on cement-based or multi-solid-waste cementitious systems [14,15,16,17,18]. Although these approaches have demonstrated the feasibility of improving mechanical performance and reducing contaminant leaching from EMR, the role of biochar in EMR stabilization remains insufficiently understood. In particular, the relationships linking SBC incorporation to Mn chemical speciation, ITZ and pore-network evolution, and Mn immobilization within the alkaline cementitious environment have not been systematically clarified. Consequently, the relative contributions of precipitation, physical encapsulation, transport limitation, and potential redox-related processes to Mn immobilization require further investigation.

Concurrent with the EMR challenge, the management of municipal sewage sludge represents another important environmental issue [19]. Thermochemical conversion of sewage sludge into biochar (SBC) through pyrolysis provides a potential valorization route, producing a carbon-rich material with a porous architecture and abundant surface functionalities [20,21,22]. In addition to its established application in pollutant adsorption, SBC has also been explored as a functional additive in cementitious composites, where it can influence hydration behavior, pore structure, and mechanical performance [23,24]. Therefore, incorporating sewage-sludge-derived biochar into EMR-based cementitious systems provides an opportunity for the simultaneous valorization of municipal and industrial solid wastes. However, the environmental implications of this coupled strategy have not been sufficiently evaluated together with its Mn immobilization performance, particularly from a life-cycle perspective.

To bridge these gaps, this study proposes an industrial-municipal symbiosis strategy integrating municipal sewage sludge-derived biochar into cement-based systems for EMR solidification. The central hypothesis is that SBC can enhance Mn immobilization through coupled microstructural and chemical effects, including pore/interfacial refinement and changes in Mn chemical speciation. Specifically, this work aims to: (i) optimize SBC pyrolysis parameters, including temperature and residence time, and SBC dosage based on the mechanical performance and Mn leaching behavior of the composites; (ii) elucidate the relationships among Mn speciation, hydration-phase evolution, ITZ/pore-network characteristics, and Mn immobilization using integrated XRD, SEM, μ-CT, XPS, and FTIR analyses; and (iii) evaluate the life-cycle environmental implications of the SBC-amended EMR solidification system. By integrating performance evaluation, multi-scale characterization, and life-cycle assessment, this study seeks to clarify the mechanisms governing Mn immobilization and assess the potential for the co-valorization of EMR and municipal sewage sludge.

## 2. Experiments

### 2.1. Raw Materials

EMR was collected from a manganese smelting plant in Hubei Province, China. Upon collection, the fresh residue was pre-dried at 45 °C to constant weight and subsequently ground into a fine powder using a ball mill. The chemical composition of EMR was determined via X-ray fluorescence (XRF) spectroscopy, as presented in Table 1. The primary oxides included SiO_2_, SO_3_, Al_2_O_3_, CaO, and MnO. Ordinary Portland cement (OPC, Grade 42.5, Huaxin Cement Co., Ltd., Huangshi, China) conforming to Chinese National Standard GB 175-2023 [25] was used as the binder. Municipal sewage sludge was sourced from the secondary sedimentation tank of a municipal wastewater treatment plant. The sludge was naturally air-dried, crushed, and passed through a 75 μm sieve prior to use.

**Figure 1:**
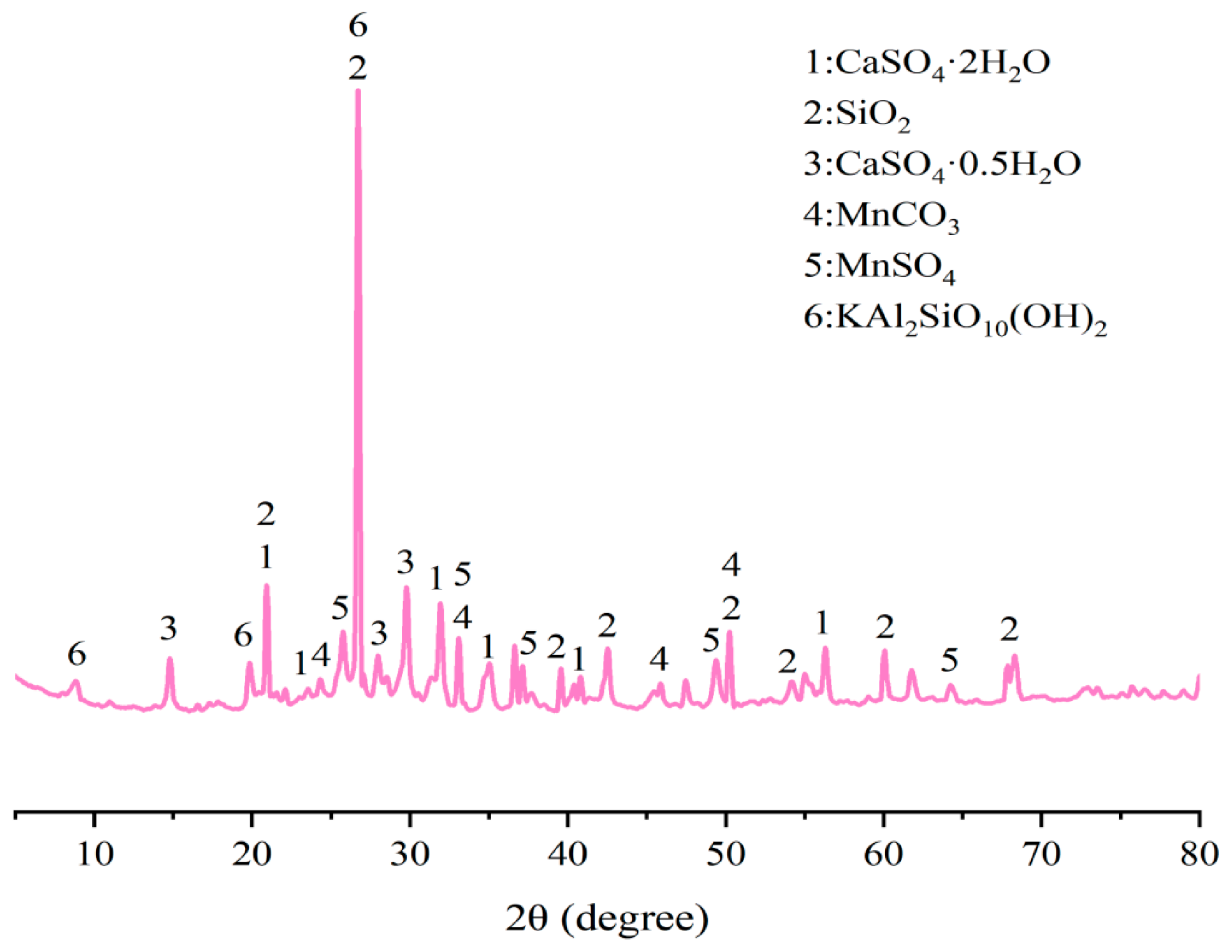
XRD patterns of the raw EMR.

Figure 1 illustrates the XRD pattern of the raw EMR. The mineralogical analysis indicates that the residue is primarily composed of quartz (SiO_2_), gypsum (CaSO_4_·2H_2_O), illite (KAl_2_Si_4_O_10_(OH)_2_), rhodochrosite (MnCO_3_), and manganese sulfate (MnSO_4_). Notably, a minor phase of hemihydrate gypsum (CaSO_4_·0.5H_2_O) was detected, likely resulting from the dehydration of dihydrate gypsum during the ball-milling process. The high solubility of Mn^2+^ in EMR is predominantly attributed to the presence of MnSO_4_.

Municipal sewage sludge was collected from the secondary sedimentation tank of a municipal wastewater treatment plant in Hubei Province, China. The wastewater treatment plant employed an anaerobic-anoxic-oxic (A^2^/O) process. The basic physicochemical properties of the raw sludge, including moisture content, pH, volatile solids (VS), and ash content, are summarized in Table 2. Its inorganic composition was further characterized by X-ray fluorescence (XRF), with SiO_2_, Fe_2_O_3_, Al_2_O_3_, P_2_O_5_, CaO, and SO_3_ identified as the major components (Table 3).

### 2.2. Preparation of SBC and Specimens

SBC was synthesized via slow pyrolysis using a vacuum tube furnace (NBD-T1700, Nobody Materials Science and Technology Co., Ltd., Zhengzhou, China) under a nitrogen atmosphere. The SBC dosage was fixed at 5 wt.% of the total dry solids during pyrolysis-condition optimization. As shown in Table 4, ECB specimens incorporating SBC prepared at 500 °C for 90 min exhibited the highest compressive strengths at 7, 14, and 28 days (2.19, 2.32, and 2.44 MPa, respectively). The pyrolysis temperature and residence time were preliminarily optimized based on the compressive strength of the resulting ECB specimens at different curing ages. According to the optimization results (Table 4), 500 °C and 90 min were selected as the optimal pyrolysis temperature and residence time, respectively. The heating rate was controlled at 10 °C/min. After pyrolysis, the biochar was cooled to room temperature, ground, and passed through a 75 μm sieve.

For the preparation of ECB specimens, EMR, OPC, SBC, and water were mixed according to the proportions listed in Table 5. The contents of EMR, OPC, and SBC were expressed as weight percentages of the total dry solids. The EMR content was fixed at 85 wt.%, while SBC was incorporated at 0, 3, 5, and 7 wt.%, respectively, by partially replacing OPC on an equal-mass basis. Accordingly, the OPC contents were 15, 12, 10, and 8 wt.%, respectively. The water-to-solid ratio was maintained at 0.30 for all mixtures. The dry components were first mixed thoroughly, subsequently water was added, and the mixture was stirred in a planetary mixer for 5 min to obtain a homogeneous slurry. The fresh mixtures were cast into 40 mm × 40 mm × 40 mm cubic molds and compacted using a vibrating table. After 24 h of curing at ambient temperature, the specimens were demolded and transferred to a standard curing chamber maintained at 20 ± 2 °C and 95% relative humidity until the designated testing ages of 7, 14, and 28 days.

### 2.3. Characterization Techniques

The mineral phases of EMR and SBC were identified using X-ray diffraction (XRD, Ultima IV, Rigaku, Akishima, Japan) with Cu Kα radiation over a 2θ range of 5–80°. The microstructure and morphology were examined using field-emission scanning electron microscopy (FE-SEM, JSM-7500F, JEOL, Akishima, Japan) operated at 5 kV. Micro-computed tomography (μ-CT, SKYSCAN 1272, Bruker microCT, Kontich, Belgium,) was employed to visualize the internal pore structure at a resolution of 5 μm. Specific surface area and pore size distribution of SBC were determined by N_2_ adsorption–desorption isotherms using a BET analyzer (Novatouch, Quantachrome, Boynton Beach, FL, USA). Fourier-transform infrared (FTIR) spectroscopy (FT-9700, PerkinElmer, Shelton, CT, USA) and X-ray photoelectron spectroscopy (XPS, AXIS Supra, Shimadzu, Kyoto, Japan) were utilized to analyze the surface functional groups and chemical states of elements.

### 2.4. Leaching Toxicity Tests

The leaching toxicity of Mn from the solidified specimens was evaluated strictly according to the Chinese standard HJ 557-2010 (Solid waste—Extraction procedure for leaching toxicity—Horizontal Vibration Method). Cured specimens were crushed and sieved through a 3 mm sieve. Deionized water was used as the leachant at a liquid-to-solid ratio of 10 L/kg. The mixtures were horizontally agitated at 110 ± 10 rpm for 8 h and subsequently allowed to stand for 16 h. The leachates were then filtered through a 0.45 μm membrane filter. The Mn concentration in the filtrate was determined using flame atomic absorption spectrometry (FAAS, AA-7000, Shimadzu, Japan) in accordance with GB/T 11911-89 [26] (Water quality—Determination of iron and manganese—Flame atomic absorption spectrophotometric method).

### 2.5. Sequential Extraction Analysis of Mn

To evaluate the chemical fraction distribution and mobility of Mn in raw EMR and solidified ECB specimens, a revised BCR sequential extraction procedure followed by aqua-regia digestion of the final residue was employed. Mn was operationally fractionated into acid-soluble/exchangeable, reducible, oxidizable, and residual fractions. The detailed extraction conditions are summarized in Table 6. After each extraction step, the suspension was centrifuged, the supernatant was collected for Mn determination, and the remaining residue was washed with deionized water before the subsequent extraction step. Mn concentrations in the extracts were determined by FAAS.

### 2.6. Strength Contribution Coefficient

To quantitatively assess the reinforcing effect of SBC on the mechanical properties of the cementitious matrix, a strength contribution coefficient (*k*) was introduced. This coefficient is defined as the ratio of the strength contribution per unit mass of SBC (*σ_m_*) to that of cement (*σ_e_*) under identical conditions. The relationship is expressed as:

*σ_m_* = k*σ_e_* (1)

Assuming the total compressive strength of the composite containing *x*% SBC (by total mass) is *σ*, the total strength can be formulated as:

*σ* = *xσ_m_* + (1 − *x*)*σ_e_* = *σ_e_*(1 − *x* + *xk*) (2)

where *σ* represents the measured compressive strength of the specimen, and *x* is the replacement ratio of SBC.

### 2.7. Carbon Footprint Assessment

A screening-level cradle-to-gate carbon footprint assessment was conducted using the treatment of 1 t of dry EMR as the functional unit. The system boundary included OPC production and the conversion of municipal sewage sludge to SBC, including sludge pretreatment/dewatering and pyrolysis. For the optimized formulation containing 85 wt.% EMR, 10 wt.% OPC, and 5 wt.% SBC, 1 t of dry EMR corresponded to 117.65 kg OPC and 58.82 kg SBC, whereas the 0 wt.% SBC reference required 176.47 kg OPC. Based on the measured SBC yield of 62.32%, production of 58.82 kg SBC required 94.39 kg of dry sludge.

Considering the high initial moisture content of sewage sludge, the energy and emissions associated with sludge pretreatment were included rather than assuming laboratory air-drying to be impact-free. Industrial-scale emission data were used to represent sludge dewatering and pyrolysis [27]. In addition, two representative sludge-incineration routes, namely direct thermal drying followed by coal co-incineration and indirect thermal drying followed by self-sustained incineration, were included as alternative management scenarios for comparison [28]. The inventory data and emission factors used for the carbon-footprint assessment are summarized in Table 7.

The global warming potential (GWP) of each scenario was calculated as:

(3) $${\mathrm{GWP}}_{S}=\sum A_{i,s}\times {\mathrm{EF}}_{i}$$

where A*_i_*_,*s*_ is the amount of material or energy input *i* in scenario *s*, and EF*_i_* is the corresponding emission factor. An OPC emission factor of 0.829 kg CO_2_-eq kg^−1^ was used, while the sludge-treatment emission factors were taken from published industrial-scale studies. Carbon-sequestration credits reported for soil application of biochar were not included in the baseline calculation because the SBC in this study was incorporated into a cementitious matrix.

## 3. Results and Discussion

### 3.1. Mechanical Performance and Its Environmental Implications

**Figure 2:**
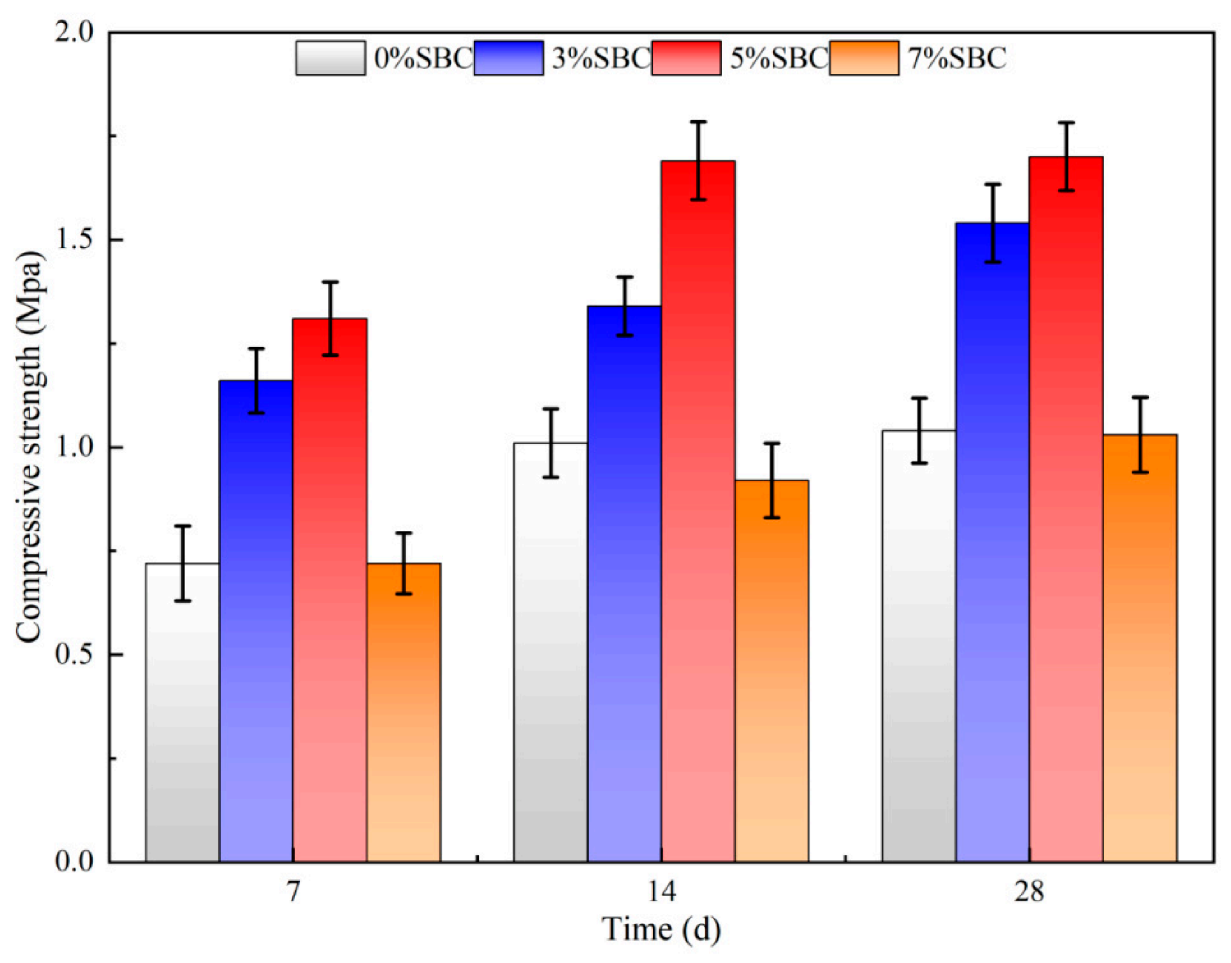
Evolution of compressive strength of ECBs with different SBC dosages at 7, 14, and 28 days.

The mechanical integrity of solidified waste forms is important for maintaining the structural stability of the matrix and limiting the potential release of encapsulated contaminants [18]. Figure 2 shows the compressive strength of ECB specimens containing different SBC dosages after 7, 14, and 28 days of curing. Compared with the control specimen (0 wt.% SBC), moderate SBC incorporation generally improved the compressive strength. Specifically, the 3 wt.% SBC specimen exhibited strength increases of 61.11%, 32.67%, and 48.08% at 7, 14, and 28 days, respectively, while the corresponding increases for the 5 wt.% SBC specimen were 81.94%, 67.33%, and 63.46%. However, further increasing the SBC dosage to 7 wt.% resulted in only limited additional strength enhancement, suggesting that excessive SBC incorporation may restrict further improvement in mechanical performance.

The strength enhancement observed at moderate SBC dosages may be associated with the porous structure and fine particle size of SBC. The SBC prepared under the optimized conditions exhibited an average pore diameter of 10.52 nm and a specific surface area of 55.06 m^2^/g. During mixing, the porous SBC particles may absorb part of the mixing water and subsequently release it during curing, thereby influencing local water availability and hydration development. In addition, the fine SBC particles (<75 μm) can occupy interparticle voids within the cementitious matrix, contributing to pore refinement and matrix densification [29,30]. These combined effects may account for the improved compressive strength observed at appropriate SBC dosages.

**Figure 3:**
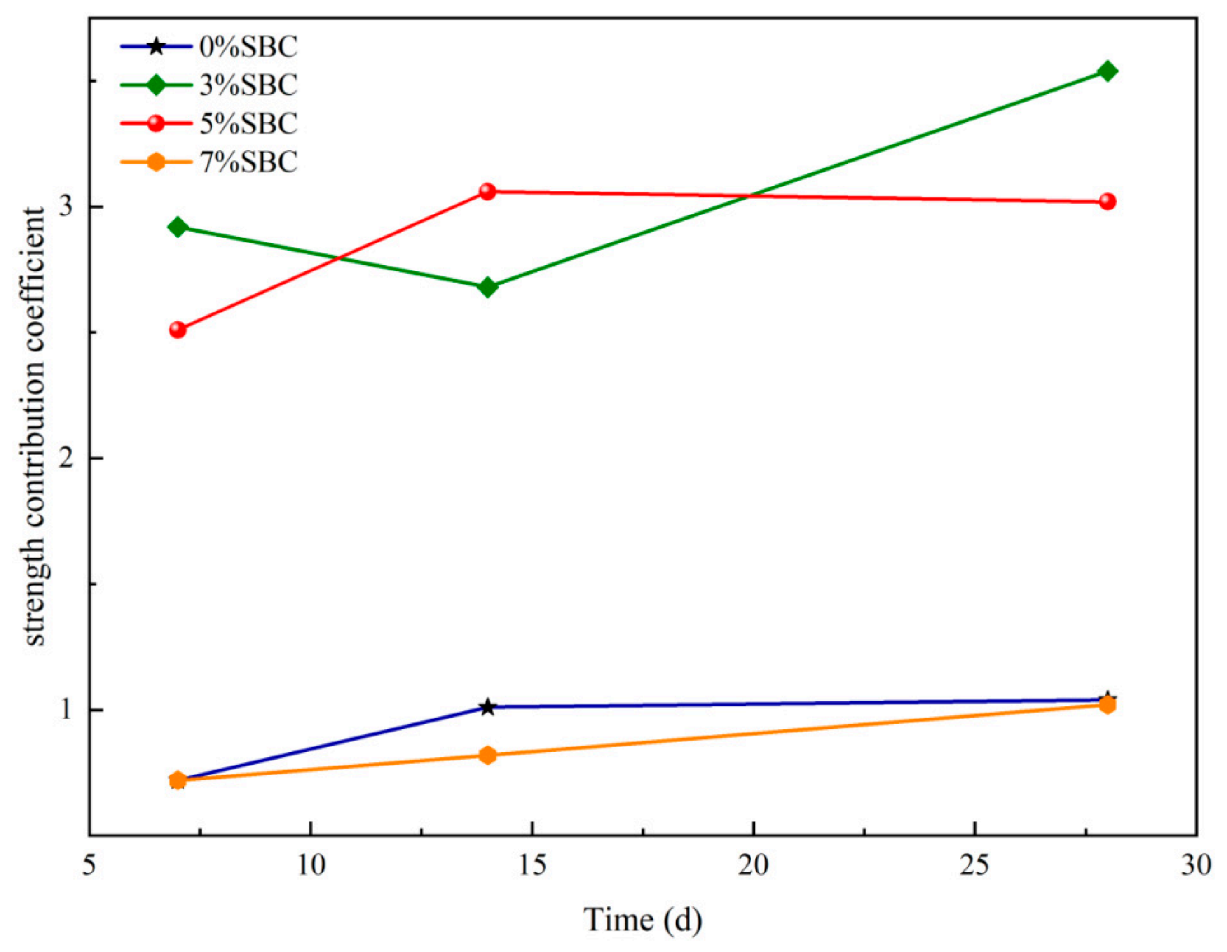
Strength contribution coefficient (*k*) of ECBs with varying SBC dosages: 0%, 3%, 5%, and 7%.

To quantitatively assess the reinforcing efficiency of SBC relative to OPC, a strength contribution coefficient (*k*) was introduced (Equations (1) and (2)), representing the ratio of strength contributed per unit mass of SBC to that of OPC. As presented in Figure 3, the *k* values varied markedly with dosage and curing age. At 7 days, the *k* value for 3% SBC (2.92) significantly exceeded that for 5% SBC (2.51). This difference may be associated with variations in effective water availability during early hydration, as higher SBC contents may retain a greater fraction of the mixing water. At 14 days, however, the 5 wt.% SBC specimen exhibited a higher *k* value (3.06) than the 3 wt.% SBC specimen (2.68). This change suggests that the contribution of SBC to strength development depends on curing age and may reflect the combined effects of water redistribution, particle filling, and continued cement hydration.

At 7 wt.% SBC, the additional contribution to strength became limited. This behavior may be associated with the increased water demand of porous biochar and the greater heterogeneity of the cementitious matrix at higher biochar contents, which may partially offset the beneficial filling and internal-curing effects observed at moderate dosages [31,32,33,34].

Overall, the 5 wt.% SBC formulation exhibited the most favorable mechanical performance among the investigated mixtures, with a 28-day compressive strength exceeding 1.5 MPa. The results indicate that partial replacement of OPC with an appropriate amount of SBC can maintain or improve the mechanical integrity of the EMR-based cementitious matrix. However, the present mechanical tests were limited to curing ages of up to 28 days and therefore do not provide direct evidence of long-term durability under field exposure conditions.

### 3.2. Optimization of SBC Pyrolysis Conditions

The pyrolysis temperature and residence time are critical parameters governing the physicochemical properties of biochar and, consequently, the mechanical performance of the EMR-based composites.

**Figure 4:**
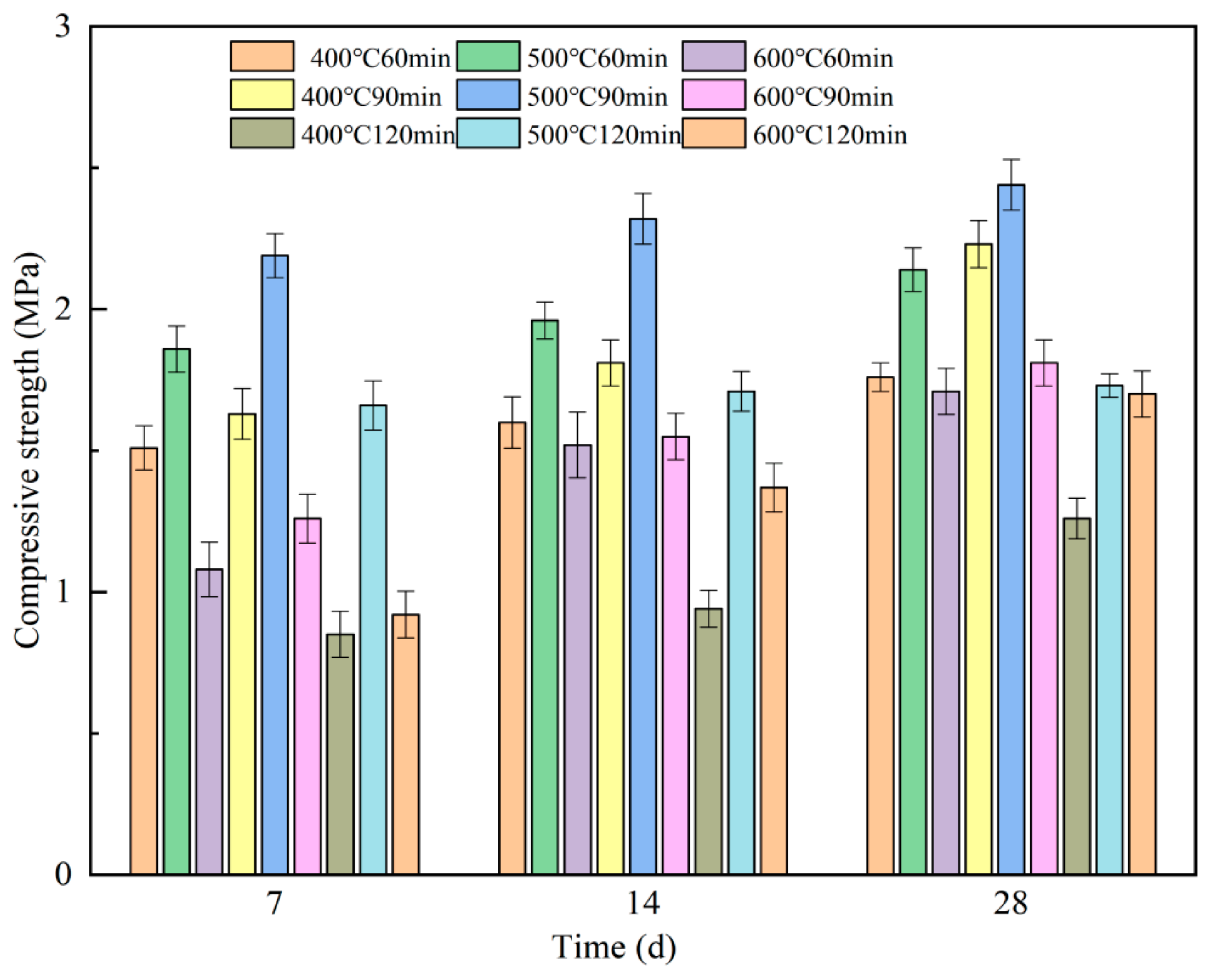
Effect of pyrolysis temperature and time on the compressive strength of ECBs at 7, 14, and 28 days.

Effect of pyrolysis temperature. As illustrated in Figure 4, the compressive strength of ECBs exhibits a typical “peak” trend with increasing pyrolysis temperature, regardless of curing age. Under a fixed pyrolysis time of 90 min, the strength increased by 34.36%, 28.18%, and 9.42% at 7, 14, and 28 days, respectively, peaking at 500 °C. This phenomenon is attributed to the structural evolution of the biochar. Within the range of 400–500 °C, the thermal decomposition of organic matter in sludge is sufficient to form a highly developed porous structure and a large specific surface area. These pores act as micro-reservoirs that absorb excess mixing water, mitigating the formation of capillary channels and reducing the initial porosity of the ECB matrix. However, exceeding 500 °C leads to a decline in strength. Excessive temperatures induce the collapse of the pore structure and the blockage of micropores due to the sintering of inorganic ash components, resulting in a reduced specific surface area and diminished internal curing capacity [23,35,36]. Additionally, low pyrolysis temperatures (<400 °C) may leave residual fatty acids and volatile organic compounds that interfere with cement hydration, hindering early strength development.

Effect of pyrolysis time. As shown in Figure 4, extending the pyrolysis duration also yielded an optimal value (90 min) for enhancing compressive strength (increases of 17.74%, 18.37%, and 14.02% at 7, 14, and 28 days, respectively, at 500 °C). Prolonged pyrolysis ensures the complete carbonization of organic matter, maximizing the porosity and water retention capacity of SBC. However, excessive residence times (>120 min) can lead to the destruction of the carbon skeleton and the consumption of volatile matter that maintains the porous architecture, ultimately degrading the physical properties of the biochar and negatively impacting the ECB strength.

### 3.3. Leaching Toxicity and Hazardous Waste Reclassification

**Figure 5:**
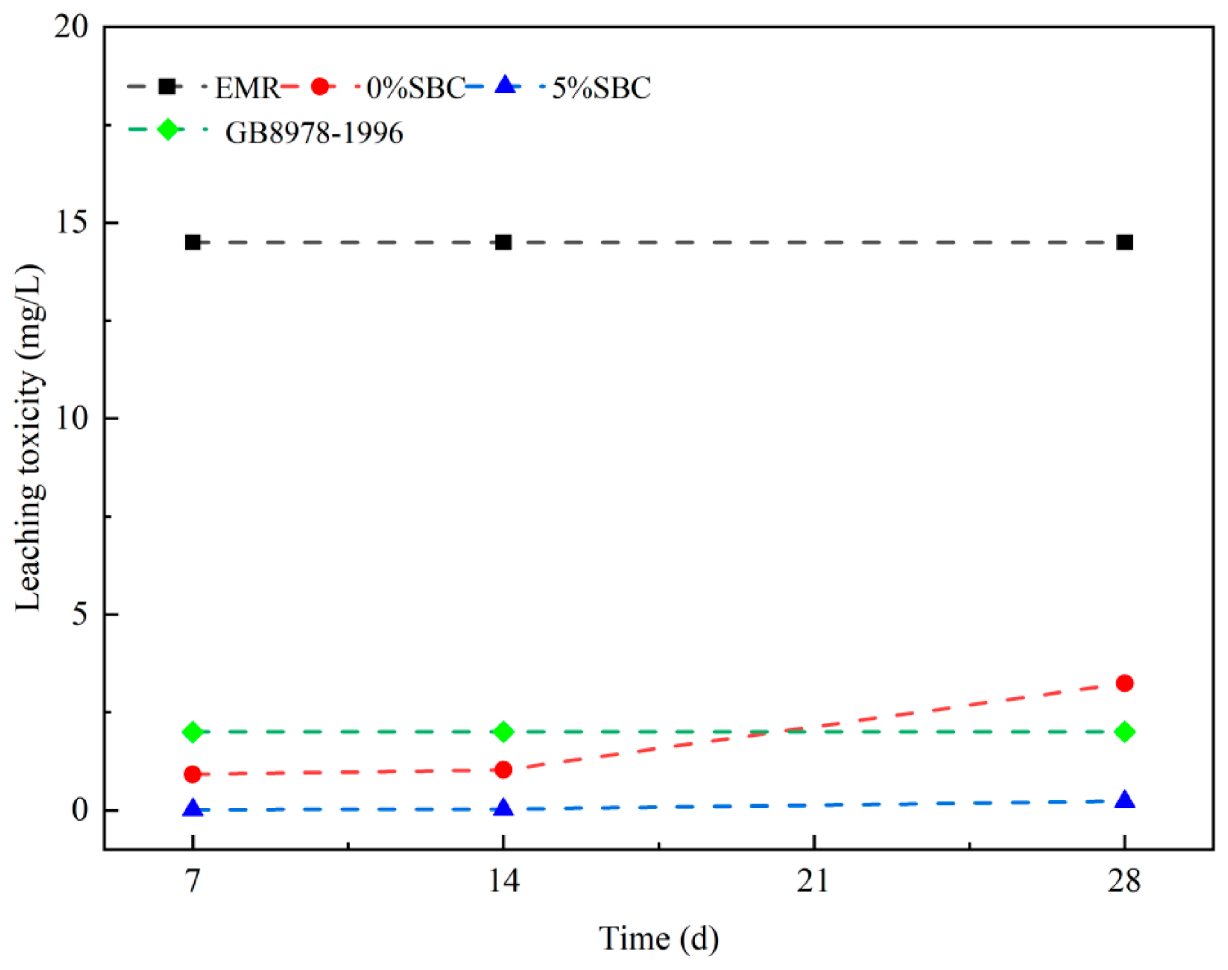
Leaching toxicity of Mn from raw EMR and cured ECBs with various dosage of SBC.

The environmental safety of solidified waste forms is ultimately governed by their ability to restrict the mobilization of hazardous constituents under field exposure conditions. Figure 5 presents the Mn leaching concentrations from raw EMR and cured ECBs as a function of curing age and SBC dosage, evaluated according to the Chinese standard HJ 557-2010 [37]. Raw EMR exhibited a high Mn leaching concentration of 14.5 mg/L, indicating high Mn mobility under the applied leaching conditions.

The incorporation of SBC induced a dramatic reduction in Mn leachability. While the control group (0% SBC) still exhibited a Mn concentration above 5 mg/L at 28 days, the optimized formulation (5% SBC, 500 °C, 90 min) reduced the Mn concentration to 0.2 mg/L at 28 days. This represents a 98.6% reduction compared to raw EMR. Under the applied test conditions, the measured value was below the relevant leaching criterion used for non-hazardous classification.

The Mn leaching concentration decreased with increasing curing age across the investigated formulations. As shown in Figure 5, the leaching concentration exhibited a monotonic decrease with extended curing age across all formulations. This trend may be associated with the progressive densification of the cementitious matrix and the formation or redistribution of Mn-bearing phases. Notably, the 5% SBC group achieved the 2 mg/L compliance threshold by 14 days and reached the near-asymptotic low level of 0.2 mg/L by 28 days, indicating that the passivation mechanism is not merely a transient surface adsorption phenomenon but rather a sustained chemical stabilization process. This distinction is critical for predicting field performance, as adsorptive immobilization is often reversible under fluctuating environmental conditions (e.g., pH changes, organic ligands), whereas the precipitation and oxidative fixation mechanisms elucidated in Section 3.5 offer greater resistance to remobilization.

Comparative analysis with previous studies further highlights the efficacy of the SBC-amended system. Previous studies have demonstrated that cementitious stabilization can substantially reduce Mn mobility and leaching from EMR through precipitation, hydration-product incorporation, and physical encapsulation [5,18]. In the present study, the Mn concentration decreased from 14.5 mg/L to 0.2 mg/L after 28 days of curing, indicating effective Mn immobilization under the tested leaching conditions. This enhanced performance is ascribed to the synergistic interplay between the porous SBC matrix and the evolving cement hydration products, as elaborated in subsequent BCR fractionation, microstructural, and spectroscopic analyses.

From an environmental perspective, the reduction from 14.5 mg/L to 0.2 mg/L indicates a substantially decreased potential for Mn release through water-mediated transport pathways. However, the present laboratory leaching test does not directly simulate long-term field exposure or groundwater transport. Therefore, the 0.2 mg/L result should be interpreted as evidence of improved Mn leaching resistance during the tested curing period rather than as direct evidence of long-term environmental safety.

### 3.4. Chemical Fraction Distribution of Mn

**Figure 6:**
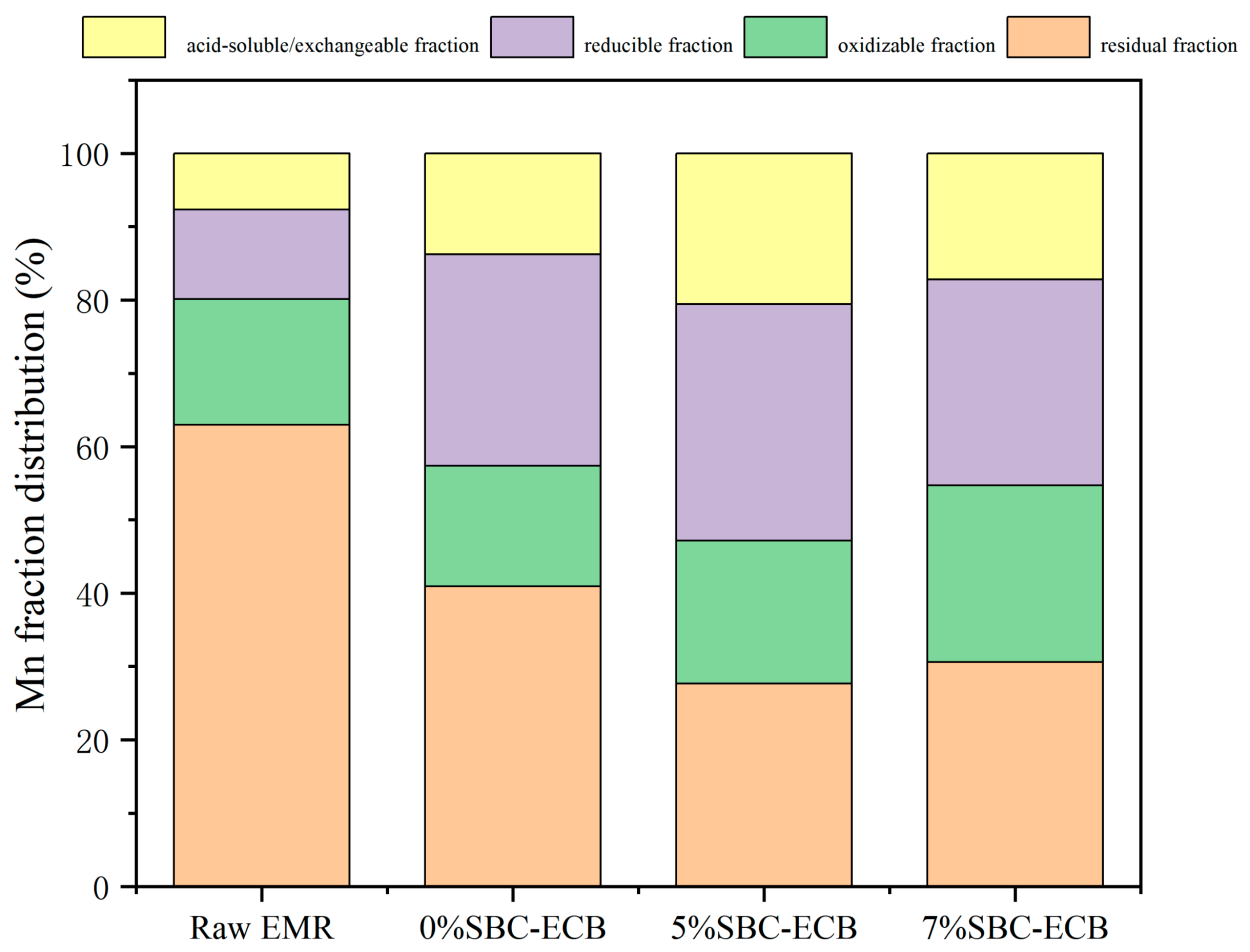
Chemical fraction distribution of Mn in raw EMR and ECB specimens with different SBC dosages.

The mobility of Mn is closely related to its chemical fraction distribution. Figure 6 shows the BCR sequential extraction results of Mn in raw EMR and ECB specimens with different SBC dosages. In raw EMR, Mn was mainly present in the acid-soluble/exchangeable fraction, accounting for 63%, followed by the reducible (17%), oxidizable (12%), and residual (8%) fractions. The predominance of the acid-soluble/exchangeable fraction indicates the relatively high mobility of Mn in raw EMR.

After cementitious solidification, the chemical fraction distribution of Mn changed markedly. The acid-soluble/exchangeable fraction decreased from 63% in raw EMR to 41%, 28%, and 31% in the 0%, 5%, and 7% SBC-ECB specimens, respectively. Meanwhile, the proportions of the oxidizable and residual fractions increased after solidification. In particular, the 5% SBC-ECB specimen exhibited the lowest proportion of acid-soluble/exchangeable Mn, while the oxidizable and residual fractions increased to 32% and approximately 21%, respectively. These results indicate that cementitious solidification promoted the redistribution of Mn from the readily mobile fraction toward more strongly bound fractions, and the incorporation of 5% SBC further enhanced this stabilization effect. When the SBC dosage increased to 7%, the acid-soluble/exchangeable fraction slightly increased to 31%, indicating that a further increase in SBC dosage did not further improve Mn stabilization.

In summary, the BCR results show that the addition of SBC promoted the redistribution of Mn toward less mobile fractions, with the 5% SBC-ECB specimen showing the most pronounced stabilization. This trend is consistent with the Mn leaching results in Section 3.3, further indicating that the decrease in Mn leachability was accompanied by changes in its chemical fraction distribution.

### 3.5. Pore Structure Characterization of SBC

The pore structure parameters of SBC prepared under optimal conditions (500 °C, 90 min) are summarized in Table 2.

**Figure 7:**
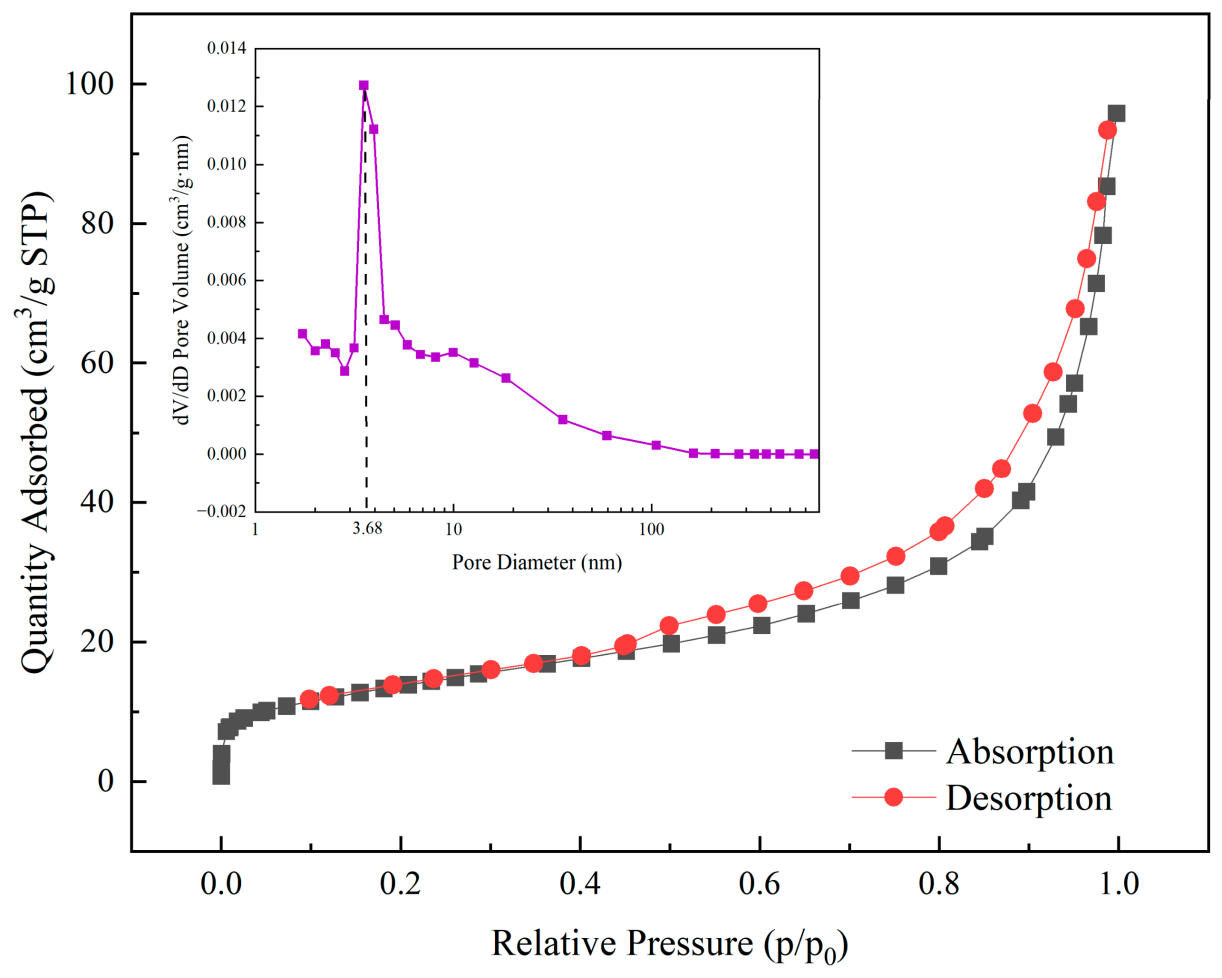
Pore size distribution (inset), N_2_ adsorption–desorption isotherms and BJH pore size distribution of SBC.

Textural properties. The N_2_ adsorption–desorption isotherm (Figure 7) exhibits a Type II profile with a distinct H3-type hysteresis loop, indicative of mesoporous materials consisting of slit-shaped pores derived from plate-like particles. The average pore diameter of SBC was determined to be 10.5 nm, classifying it as a mesoporous material. Notably, SBC prepared under the optimized conditions (500 °C, 90 min) yielded a specific surface area of 55.06 m^2^/g, markedly surpassing that of samples obtained under alternative conditions.

Correlation with mechanical performance. The high specific surface area and mesoporous structure provide a dual mechanism for strength enhancement:

1. Internal curing: The porous network absorbs free water during mixing and releases it gradually to sustain cement hydration, explaining the continuous strength gain observed at later ages (14–28 days).
2. Micro-aggregate effect: With a particle size finer than cement (D_50_ < 75 μm), SBC particles fill the interstitial voids within the matrix, densifying the microstructure and reducing the porosity at the ITZs (Interfacial Transition Zones).

Mechanism of pore formation. The superior textural properties at 500 °C are attributed to the complete devolatilization of organic components. As pyrolysis progresses, the escaping gases (CO_2_, CO, CH_4_) create new channels and pores within the carbonaceous matrix, increasing the specific surface area (SSA) [35]. However, as noted in Section 3.2, temperatures beyond 500 °C cause the thermal rearrangement of carbon layers and ash sintering, which collapses the pore walls and reduces the SSA. This relationship between the textural properties of SBC and the compressive strength of ECB validates the selection of 500 °C and 90 min as the optimal preparation parameters [38,39].

### 3.6. Microstructure and Composition Analysis

The exceptional reduction in Mn leachability (98.6%) and enhanced mechanical performance of SBC-amended ECBs are rooted in a synergistic immobilization mechanism combining physical encapsulation within a low-permeability hydration matrix and chemical stabilization via speciation transformation of Mn. A suite of complementary characterization techniques was employed to deconvolute these mechanisms across length scales, from macroscopic pore networks to atomic-level chemical bonding.

#### 3.6.1. Phase Evolution and Mineralogical Analysis (XRD)

**Figure 8:**
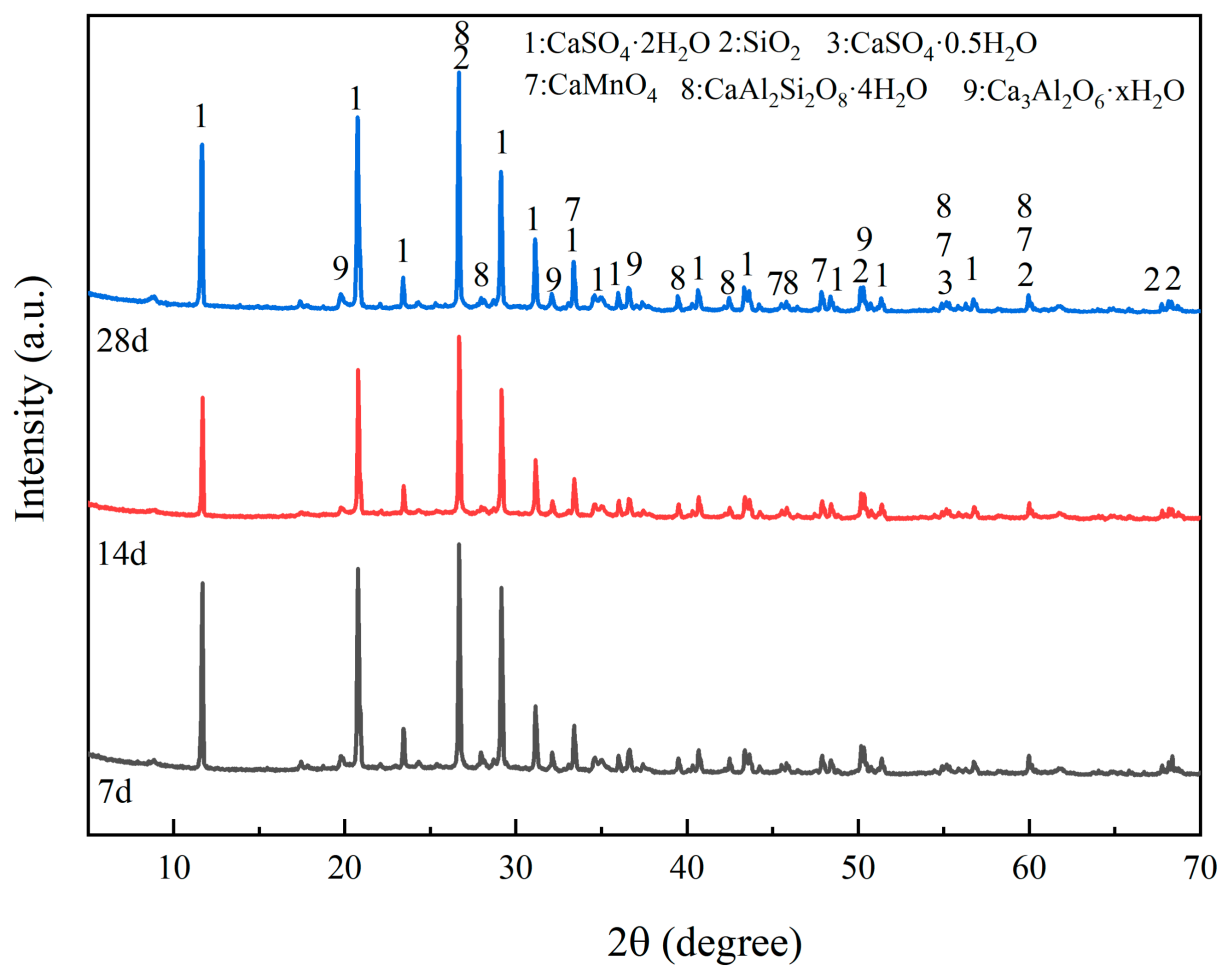
XRD patterns of ECBs cured for 7, 14 and 28 days.

XRD analysis (Figure 8) revealed the mineralogical transformations underpinning Mn immobilization. The disappearance of anhydrite (CaSO_4_) peaks from raw EMR and the concomitant emergence of strong gypsum (CaSO_4_•2H_2_O) and ettringite (AFt, Ca_6_Al_2_(SO_4_)_3_(OH)_12_•26H_2_O) reflections indicate the complete consumption of anhydrous sulfates during hydration. The formation of AFt—a voluminous hydration product that expands to fill interstitial voids—may contribute to matrix densification and consequently restrict Mn transport through the cementitious matrix.

Although crystalline Mn-bearing phases could not be unambiguously identified by XRD in 28-day ECBs, the possible formation of manganese oxides cannot be excluded. The alkaline environment suggests that Mn precipitation may contribute to its immobilization within the cementitious matrix. Such precipitation may represent a possible chemical pathway for Mn stabilization, together with physical encapsulation and other possible interactions within the hydration matrix. The increasing intensity of AFt and C-S-H peaks with curing age further corroborates the progressive densification of the matrix, aligning with the monotonic decrease in Mn leaching observed in Section 3.3.

#### 3.6.2. Microstructural Densification and Interfacial Reinforcement (SEM)

**Figure 9:**
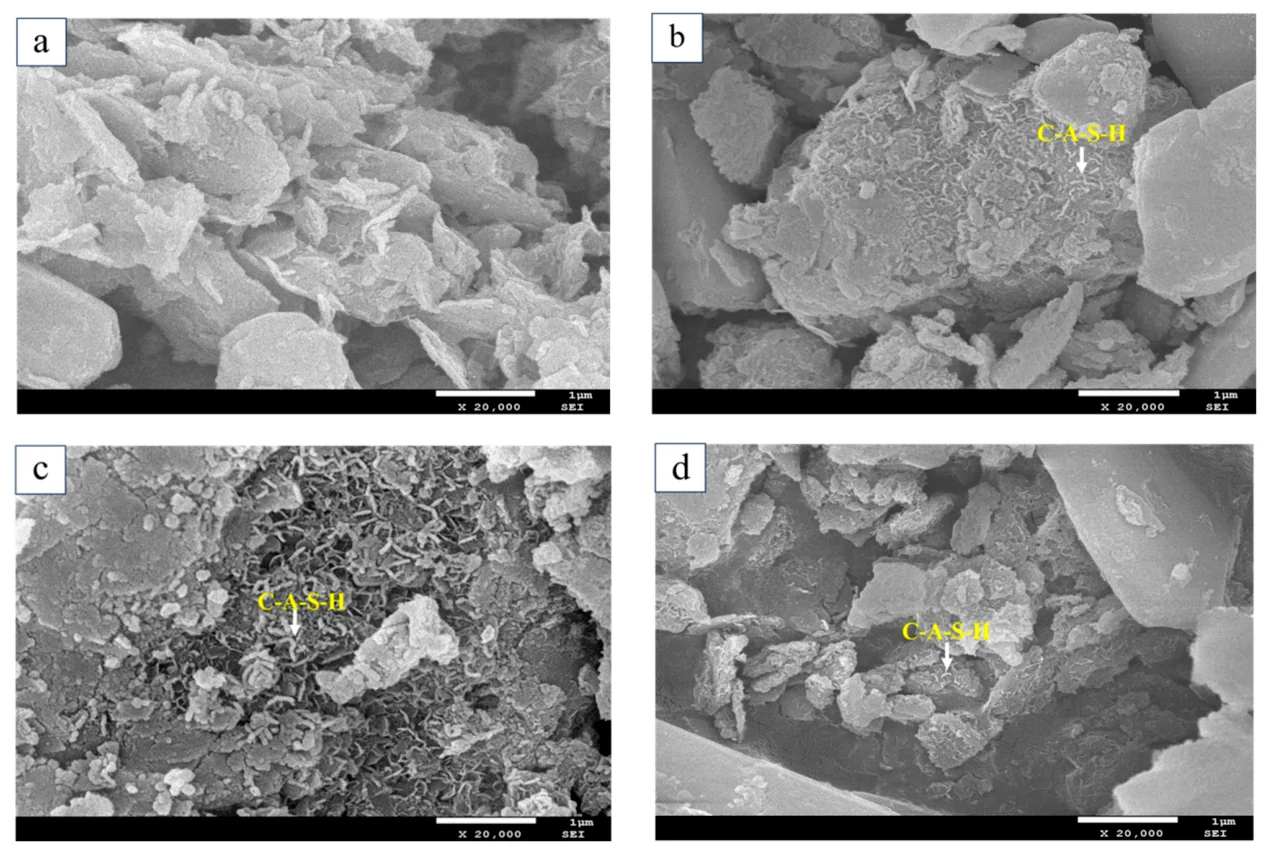
SEM micrographs of ECBs prepared with SBC at 28 days: (**a**) 0% dosage; (**b**) 400 °C for 90 min; (**c**) 500 °C for 90 min; (**d**) 600 °C for 90 min.

SEM imaging (Figure 9) elucidated the microstructural basis for the enhanced mechanical and environmental performance. The control group (0% SBC) exhibited a loosely packed matrix with abundant microcracks and weakly bonded interfacial transition zones (ITZs) surrounding unreacted EMR particles, which may provide relatively favorable pathways for fluid penetration and Mn migration. In contrast, SBC-modified ECBs displayed a highly compact microstructure characterized by interwoven fibrous C-S-H gels and needle-like AFt crystals that formed denser hydration network surrounding both SBC and EMR particles [30,31].

**Figure 10:**
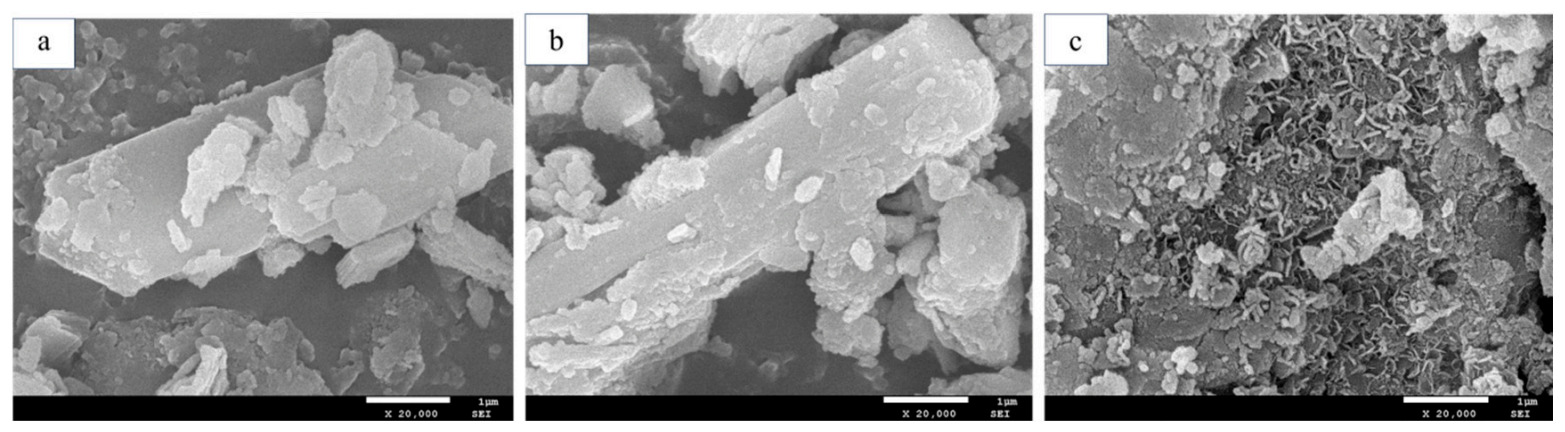
Microstructural evolution of ECBs at (**a**) 7 days, (**b**) 14 days, and (**c**) 28 days.

The fine SBC particles (<75 μm) may provide additional surfaces for the nucleation and growth of hydration products. C-S-H gels were observed in close association with the rough and porous surfaces of SBC, suggesting improved interfacial contact between SBC particles and the surrounding cementitious matrix. This interfacial reinforcement not only enhanced compressive strength (Section 3.1) but also may contribute to the reduction of connected microcracks and pore continuity. As shown in Figure 10, the maturity of this microstructure at 28 days—with near-complete coverage of EMR surfaces by C-S-H is consistent with the lower Mn leaching concentration observed at 28 days and suggests that matrix densification contributed to restricting Mn transport.

#### 3.6.3. Speciation Transformation and Chemical Bonding (XPS and FTIR)

**Figure 11:**
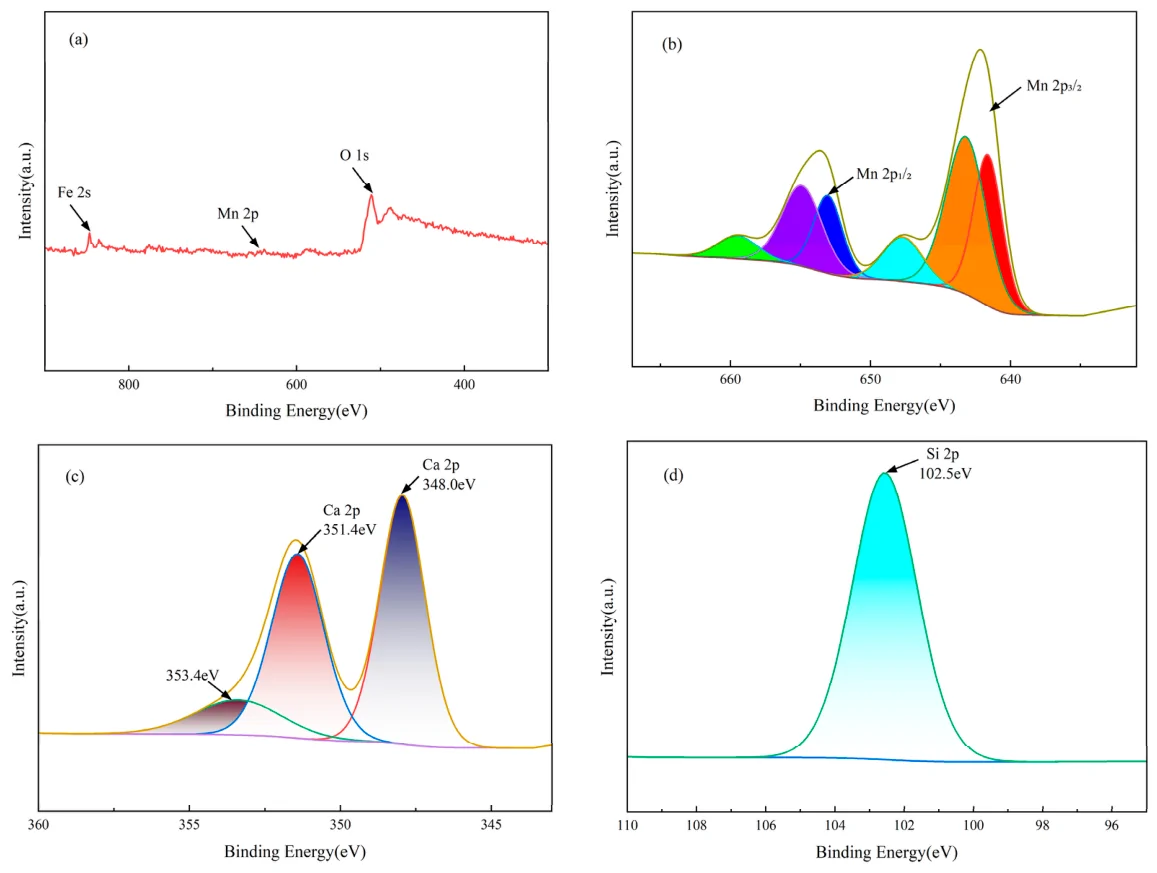
High-resolution XPS spectra of raw EMR: (**a**) survey scan; (**b**) Mn 2p; (**c**) Ca 2p; (**d**) Si 2p. The differently colored shaded areas in (**b**–**d**) represent individual peak-fitting components, while the overlaid solid curves represent the overall fitted spectra.

**Figure 12:**
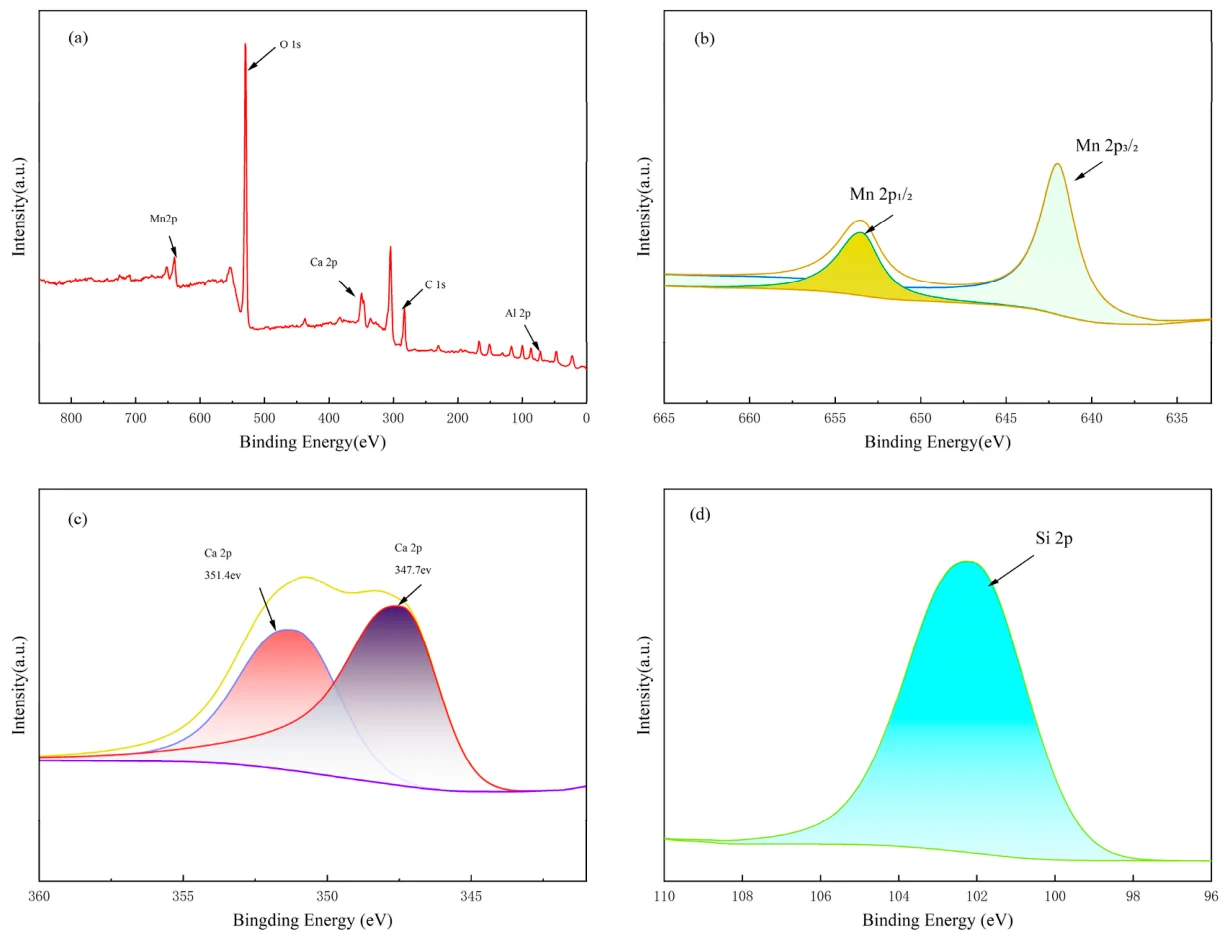
XPS spectra of ECB without SBC: (**a**) survey scan; (**b**) Mn 2p; (**c**) Ca 2p; (**d**) Si 2p. The differently colored shaded areas in (**b**–**d**) represent individual peak-fitting components, while the overlaid solid curves represent the overall fitted spectra.

**Figure 13:**
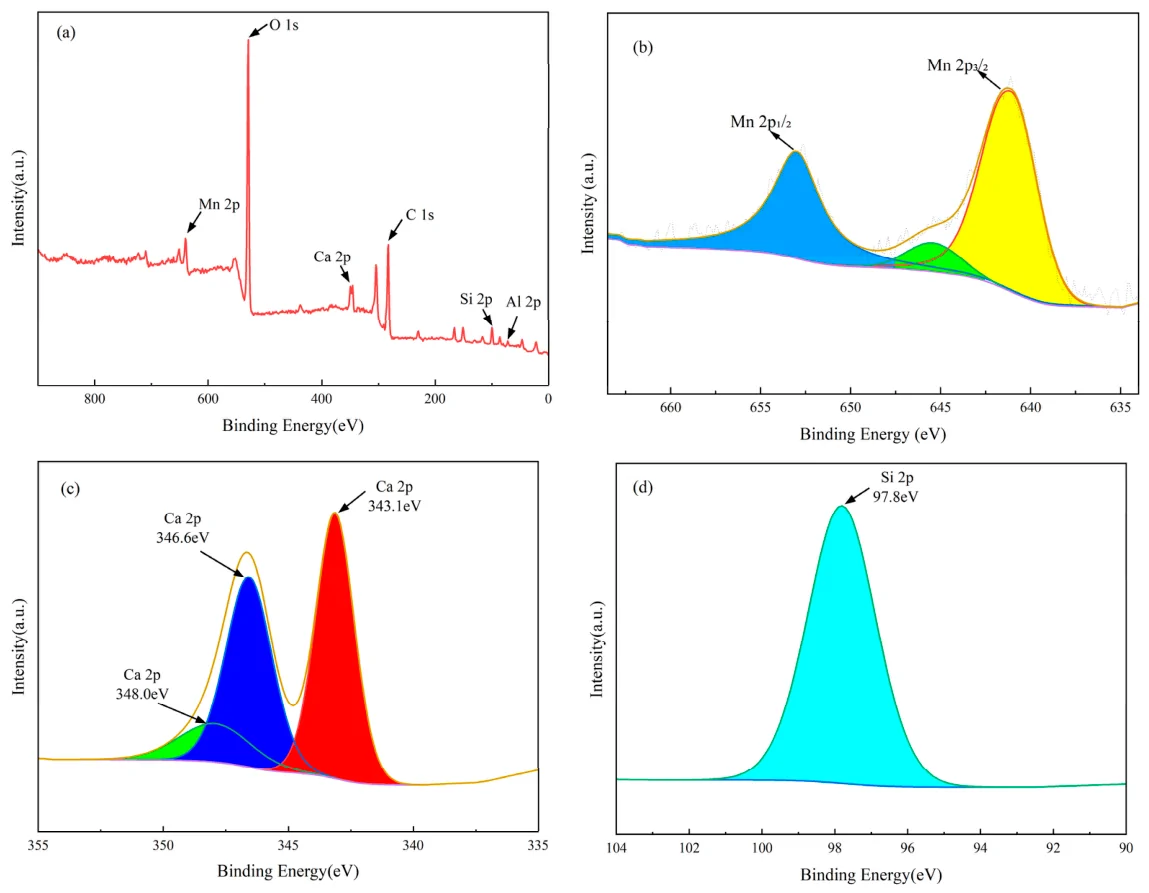
XPS spectra of ECB containing 5% SBC: (**a**) survey scan; (**b**) Mn 2p; (**c**) Ca 2p; (**d**) Si 2p. The differently colored shaded areas in (**b**–**d**) represent individual peak-fitting components, while the overlaid solid curves represent the overall fitted spectra.

High-resolution XPS analysis provided atomic-level insight into the chemical fate of Mn during hydration (Figure 11, Figure 12 and Figure 13). The survey spectrum of ECB without SBC exhibited characteristic signals of O, C, Si, Al, Ca, and Mn. In the high-resolution Mn 2p spectrum, two characteristic peaks were observed at approximately 642.0 and 653.6 eV, corresponding to the Mn 2p_3/2_ and Mn 2p_1/2_ spin–orbit components, respectively. The relatively broad Mn 2p_3/2_ peak indicates that Mn was present in heterogeneous local chemical environments after cementitious solidification. Meanwhile, the Ca 2p spectrum exhibited characteristic peaks at approximately 347.7 and 351.4 eV, assigned to Ca 2p_3/2_ and Ca 2p_1/2_, respectively, while the Si 2p peak was centered at approximately 102.5 eV. These Ca- and Si-related signals are consistent with the formation of Ca–Si-containing hydration products during cement hydration. Together with the XRD and SEM results, the XPS analysis indicates that cement hydration altered the surface chemical environment of Mn and provided a hydrated matrix favorable for Mn retention.

Further evidence for chemical immobilization came from the Si 2p and Ca 2p spectra (Figure 11c,d, Figure 12c,d and Figure 13c,d). The leftward shift of Si 2p and Ca 2p binding energies in ECBs relative to raw EMR indicates a reduction in the polymerization degree of Si–O and Ca–O bonds, corresponding to the depolymerization of crystalline silicates in EMR and their repolymerization into amorphous C-S-H gels. The peak at 97.8 eV in the Si 2p spectrum is characteristic of Q^0^ bridging tetrahedra in C-S-H, which can incorporate Mn^2+^ into the silicate framework via isomorphous substitution, providing an additional layer of chemical stabilization.

**Figure 14:**
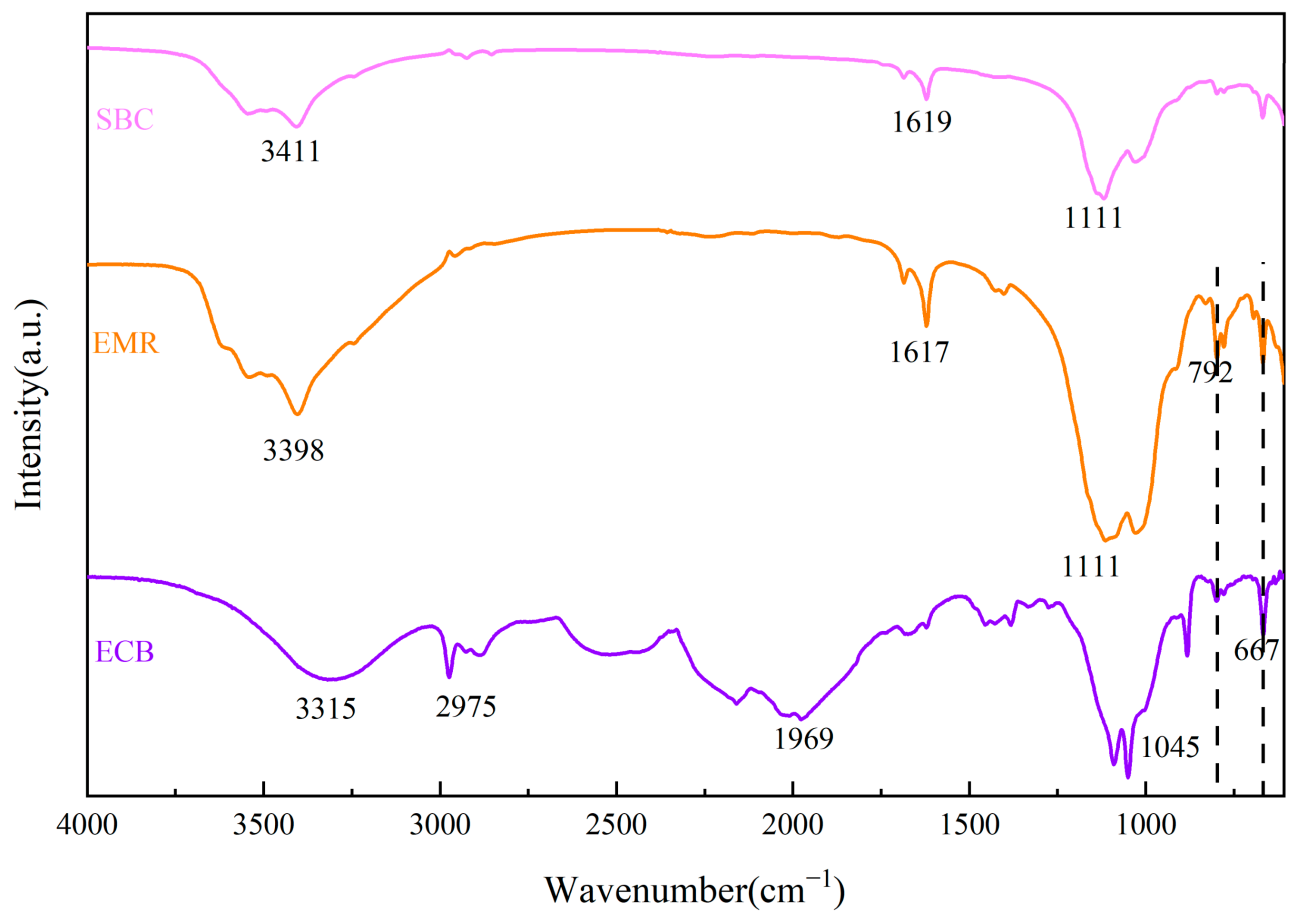
FTIR spectra of SBC, raw EMR and ECB.

FTIR spectroscopy (Figure 14) corroborated these findings. The broadening and redshift of the O–H stretching band from 3398 cm^−1^ in EMR to approximately 3300 cm^−1^ in ECB indicate enhanced hydrogen bonding associated with the formation of hydration products. The asymmetric stretching vibration of Si-O bonds appeared at approximately 1045 cm^−1^, which is consistent with the formation of hydrated silicate phases in the cementitious matrix. In addition, the band at approximately 792 cm^−1^ in EMR shifted to around 667 cm^−1^ after solidification, suggesting changes in the local metal-oxygen bonding environment. These spectral variations indicate that Mn immobilization was accompanied by changes in the surrounding chemical and bonding environment within the hydrated matrix, together with the physical retention provided by the solidified structure.

#### 3.6.4. Pore Network Tortuosity and Transport Limitation (CT)

**Figure 15:**
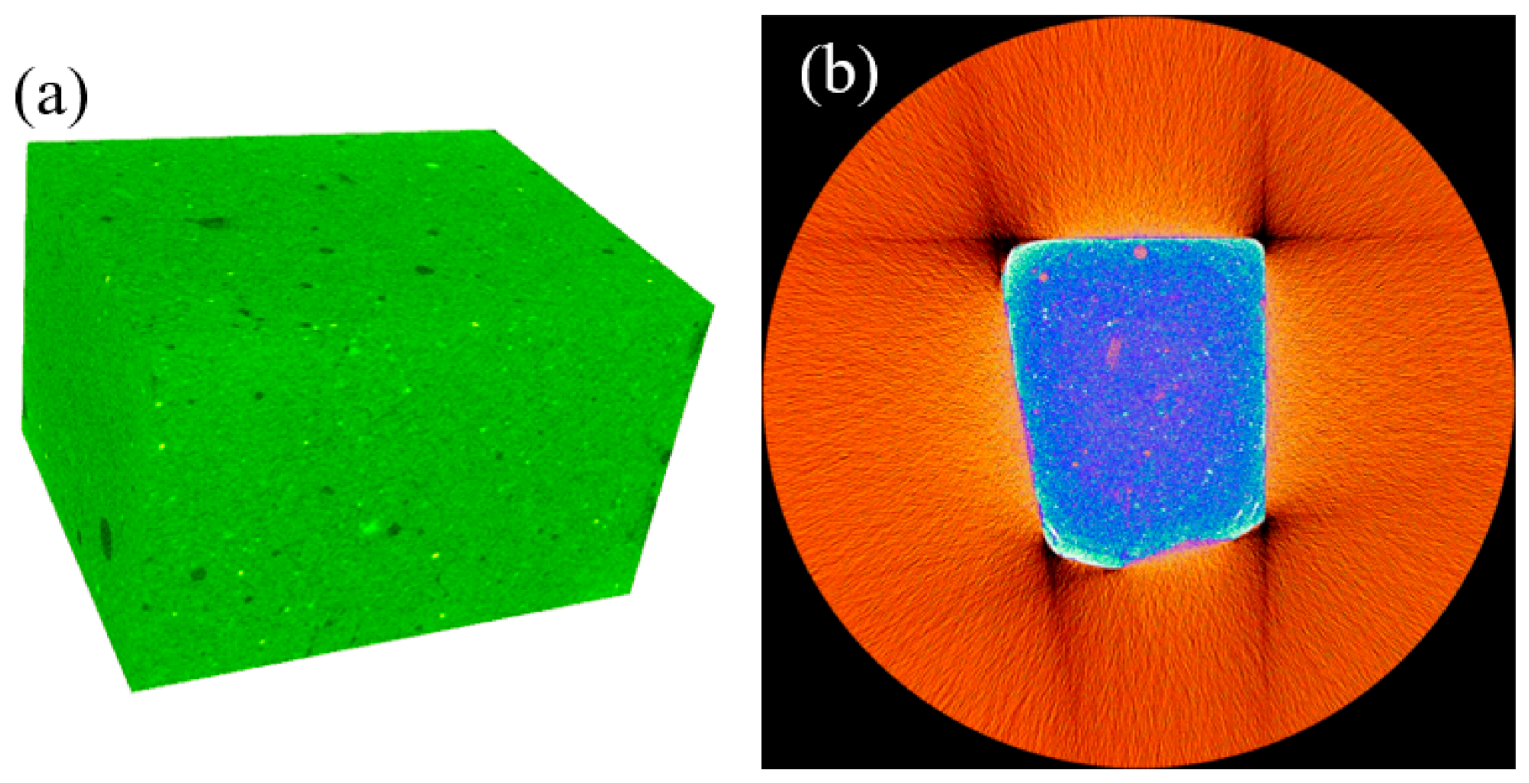
3D reconstruction and cross-sectional CT images of the ECB microstructure: (**a**) 3D reconstructed image; (**b**) cross-sectional CT image.

Micro-CT visualization (Figure 15) provided non-destructive quantification of the pore network architecture governing Mn transport. The 3D reconstructed volumes revealed that SBC particles were uniformly dispersed throughout the matrix, occupying interstitial pores and reducing the total porosity by ~28% relative to the control group. Crucially, the incorporation of SBC disrupted the continuity of capillary channels, increasing the tortuosity (*τ*) of the pore network from 2.1 (0% SBC) to 4.7 (5% SBC). Tortuosity is a fundamental parameter controlling diffusive transport: the effective diffusion coefficient (Deff) scales with 1/*τ*^2^, meaning the 4.7-fold increase in τ reduced the Mn diffusion rate by ~22-fold. This quantitative relationship directly explains the 98.6% reduction in Mn leaching: even if trace Mn remains mobile within the pore solution, its release to the environment is kinetically limited by the highly tortuous transport pathways.

#### 3.6.5. Integrated Immobilization Mechanism

**Figure 16:**
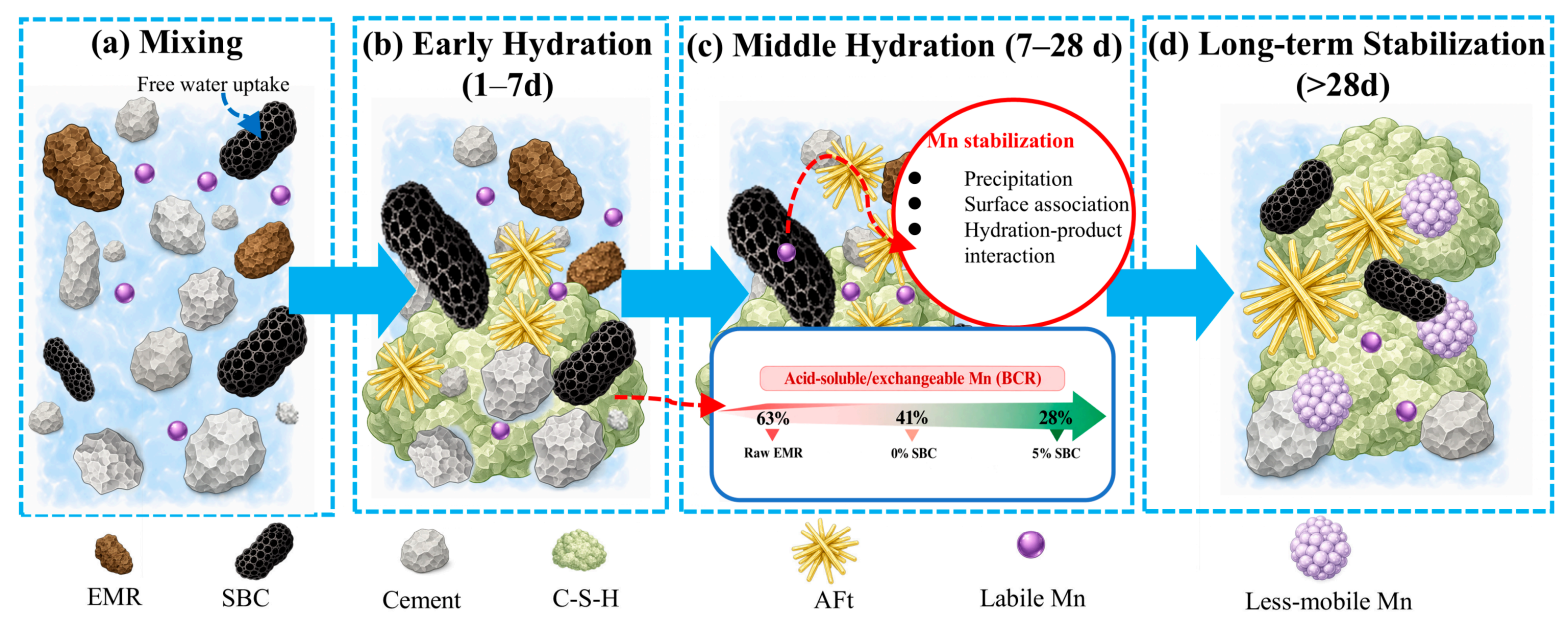
Schematic illustration of the four-stage sequential Mn immobilization mechanism in ECBs: (**a**) mixing stage; (**b**) early hydration (1–7 d); (**c**) middle hydration (7–28 d); (**d**) long-term stabilization (>28 d).

Synthesizing the multi-scale evidence, the Mn immobilization process follows a four-stage sequence (Figure 16, schematic):

1. Mixing stage: SBC absorbs excess free water, mitigating the formation of continuous capillary channels and reducing initial matrix porosity.
2. Early hydration (1–7 days): SBC releases absorbed water to sustain cement hydration, promoting the formation of C-S-H and AFt that progressively surround EMR particles and contribute to Mn retention within the cementitious matrix.
3. Middle hydration (7–28 days): With the continued development of hydration products, Mn is progressively stabilized through precipitation, surface association, and interaction with the hydrated matrix. Meanwhile, SBC modifies the interfacial environment and further promotes the redistribution of Mn toward less mobile fractions, as supported by the BCR results.
4. Long-term stabilization (>28 days): The matured, low-permeability matrix with high tortuosity blocks advective transport pathways, and the chemically fixed Mn phases remain thermodynamically stable even under environmental perturbations.

This dual physical–chemical mechanism enhances Mn immobilization through precipitation, surface association, interaction with hydration products, and physical confinement within the densified matrix. The decrease in Mn leaching during the 7–28 day curing period demonstrates improved leaching resistance under the tested conditions, whereas the long-term stability of immobilized Mn under variable environmental conditions requires further investigations.

### 3.7. Environmental Sustainability, Carbon Mitigation, and Policy Implications

The technological efficacy of the SBC-amended co-valorization system—demonstrated by the 98.6% reduction in Mn leaching and >1.5 MPa mechanical strength—translates into tangible environmental, climatic, and regulatory benefits. This section synthesizes these multidimensional advantages to elucidate the broader sustainability implications of scaling this approach for bulk EMR management.

#### 3.7.1. Regulatory Implications and Potential Economic Benefits

Under China’s relevant standards for solid-waste management, Mn leaching behavior is an important indicator for evaluating the environmental performance of treated EMR. Raw EMR exhibited a Mn leaching concentration of 14.5 mg/L. In contrast, the optimized SBC-amended ECB achieved a Mn leaching concentration of 0.2 mg/L after 28 days of curing, indicating that the treated material met the relevant Mn leaching criterion under the tested conditions. This substantial reduction in Mn leachability provides an important basis for further regulatory assessment of the treated EMR; however, compliance with the Mn criterion alone does not constitute formal hazardous-to-non-hazardous waste reclassification.

This improved leaching performance may provide potential economic benefits if the treated material satisfies all applicable regulatory requirements. For a typical manganese smelter producing 100,000 tonnes of EMR annually, reduced requirements for specialized treatment and disposal could potentially decrease waste-management costs and improve the economic feasibility of EMR treatment. Moreover, reduced Mn leachability may reduce the management burden associated with EMR and expand potential resource-utilization options, subject to compliance with relevant environmental and product-quality standards. These benefits could extend beyond individual facilities by reducing the pressure associated with large-scale EMR storage and disposal in major manganese-producing regions.

#### 3.7.2. Carbon Footprint Mitigation: Quantified Climate Co-Benefits

Beyond the environmental performance of the stabilized material itself, the revised carbon-footprint assessment further examined the climate implications of the SBC-amended route. Based on the updated inventory in Section 2.6, the 5 wt.% SBC system showed a GWP of 249.2 kg CO_2_-eq per tonne of dry EMR. The result can be interpreted from three main contributions:

1. Cement displacement (48.8 kg CO_2_-eq saving).

Incorporation of 5 wt.% SBC reduced OPC consumption from 176.47 to 117.65 kg per tonne of dry EMR. Based on an OPC emission factor of 0.829 kg CO_2_-eq t^−1^, this replacement avoided approximately 48.8 kg CO_2_-eq.

2. Sludge-to-SBC processing (151.7 kg CO_2_-eq).

Production of 58.82 kg SBC required 94.39 kg of dry sewage sludge based on the measured SBC yield of 62.32%. Using the industrial-scale emission factor for sludge dewatering and pyrolysis, this process contributed approximately 151.7 kg CO_2_-eq t per tonne of dry EMR.

3. Alternative sludge-management scenarios.

When the same amount of sewage sludge was assumed to be treated by direct thermal drying followed by coal co-incineration, the total GWP of the reference system was approximately 253.3 kg CO_2_-eq t^−1^ EMR. In contrast, indirect thermal drying followed by self-sustained incineration resulted in a lower GWP of approximately 179.4 kg CO_2_-eq t^−1^ EMR. These results show that the relative climate benefit of the SBC route depends strongly on the counterfactual sludge-management pathway.

Overall, the revised analysis indicates that the SBC route does not provide a fixed GWP reduction under all scenarios. Its climate performance is closely related to the energy demand for sludge drying and pyrolysis, auxiliary fuel consumption, and energy recovery efficiency. Therefore, further improvement in the energy efficiency of SBC production is important for enhancing the environmental competitiveness of the proposed co-valorization strategy.

#### 3.7.3. Industrial-Municipal Symbiosis: Addressing Dual Waste Challenges

The co-valorization strategy exemplifies industrial-municipal symbiosis, simultaneously addressing two concurrent waste management crises in manganese-producing regions:

Industrial waste (EMR): >150 Mt stockpiled nationally, growing at ~1.5 Mt/year, with limited disposal options due to hazardous classification.

Municipal waste (sewage sludge): >90 Mt/year nationally by 2025, overwhelming landfilling and incineration capacities.

By integrating SBC production into EMR solidification, this approach colocates the treatment of both waste streams at the intersection of manganese smelters and municipal wastewater treatment plants (WWTPs). This spatial synergy eliminates dual transportation burdens, reduces capital expenditure on standalone sludge pyrolysis facilities, and creates a closed-loop material flow wherein the “waste” from one sector (municipal sludge) becomes a value-added resource for another (EMR stabilization). Such symbiotic configurations are increasingly recognized by the UN Environment Programme as a cornerstone of sustainable waste management in developing economies.

#### 3.7.4. Policy Relevance and Implementation Roadmap

The findings of this study carry direct implications for waste management policymaking in manganese-producing jurisdictions worldwide:

For regulators: The quantified 0.2 mg/L Mn leaching threshold provides a defensible technical basis for revising EMR management protocols, potentially allowing conditionally stabilized EMR to bypass hazardous waste classification pending third-party verification. This would alleviate the regulatory bottleneck constraining the manganese industry while maintaining environmental safeguards.

For industry: The dual benefits of cost reduction (landfill savings) and carbon mitigation (GWP reduction) create a compelling business case for voluntary adoption, even absent regulatory mandates. Early adopters could leverage the technology for green financing opportunities under China’s expanding green bond market.

For researchers: The oxidative immobilization mechanism mediated by SBC redox functionalities opens new avenues for designing biochar-based stabilizers tailored to other redox-sensitive heavy metals (e.g., Cr^6+^, As^3+^, Se^4+^), broadening the applicability of this approach beyond manganese residues.

#### 3.7.5. Limitations and Future Research Directions

While the laboratory-scale results demonstrate exceptional promise, several aspects warrant field-scale validation prior to full commercial deployment:

1. Long-term durability: Accelerated aging tests (freeze–thaw cycles, wet–dry alternation, carbonation exposure) are needed to confirm that the 0.2 mg/L leaching threshold is maintained over decadal timescales under real-world weathering conditions.
2. Complex leachate chemistry: Real EMR leachates contain coexisting anions (SO_4_^2−^, Cl^−^, NH_4_^+^) and organic ligands that may compete with Mn^2+^ for adsorption/precipitation sites or form soluble complexes. Column leaching tests simulating multi-decade percolation are recommended.
3. Techno-economic feasibility at scale: Pilot-scale trials (~1000 tonne demonstrations) are necessary to validate the energy efficiency of SBC production, the homogeneity of large-scale ECB mixing, and the actual cost savings under industrial conditions.

Addressing these gaps in future work will solidify the transition from laboratory proof-of-concept to engineered reality, ultimately contributing to the United Nations Sustainable Development Goals—particularly SDG 9 (Industry Innovation), SDG 11 (Sustainable Cities and Communities), SDG 12 (Responsible Consumption and Production), and SDG 13 (Climate Action).

## 4. Conclusions

This study demonstrates the synergistic physical–chemical immobilization of Mn in EMR co-solidified with sewage sludge-derived biochar (SBC), establishing a technically viable pathway for dual-waste valorization. The key conclusions are summarized as follows:

1. Triple functional roles of SBC in alkaline cementitious systems: Beyond physical micro-aggregate filling and internal curing, SBC modifies the hydration/interfacial environment and promotes the redistribution of Mn toward less mobile fractions. BCR analysis showed that the acid-soluble/exchangeable Mn fraction decreased from 63% in raw EMR to 41% in ECB without SBC and further to 28% after the incorporation of 5 wt% SBC. These results indicate that cementitious solidification provides the primary stabilization environment, while SBC further enhances Mn immobilization through interfacial regulation and Mn fraction redistribution.
2. Quantified structure–performance relationship: The incorporation of 5 wt% SBC increases the pore network tortuosity by 124% (from 2.1 to 4.7) and eliminates weak interfacial transition zones (ITZs) around EMR particles. This microstructural refinement kinetically restricts Mn diffusion, reducing Mn leaching from 14.5 mg/L (raw EMR) to 0.2 mg/L—a 98.6% reduction after 28 days of curing. The resulting Mn concentration was below the relevant leaching criterion for non-hazardous classification under the tested conditions. The 28-day compressive strength of the optimized formulation (>1.5 MPa) demonstrates satisfactory mechanical integrity.
3. Quantified sustainability benefits: Life-cycle assessment demonstrated the potential of the SBC-amended system to reduce global warming potential relative to the reference treatment scenario, primarily through reduced OPC consumption and the beneficial utilization of sewage sludge. The magnitude of the environmental benefit depends on the sludge pretreatment and thermal treatment pathway considered in the assessment.
4. Generalizable immobilization mechanism: The four-stage synergistic process—water absorption during mixing, physical encapsulation during early hydration, progressive Mn stabilization through precipitation, surface association, and interaction with the hydrated matrix during middle hydration, and transport limitation through the densified and highly tortuous pore network-provides a mechanistic framework for understanding Mn immobilization in SBC-amended cementitious systems. SBC further enhances this process by regulating the hydration/interfacial environment, promoting Mn redistribution toward less mobile fractions, and refining the pore structure.

Forward-looking implications: While laboratory-scale results demonstrate the potential of this approach, its performance under complex leachate conditions requires further field-scale evaluation. Future work should also optimize SBC pyrolysis parameters at pilot scale to reduce production costs, supporting the transition from laboratory proof-of-concept to industrial implementation. This study offers a defensible engineering basis for the co-management of EMR and municipal sludge, contributing to sustainable solid waste valorization in manganese-producing regions worldwide.

## Figures and Tables

**Table 1:** Major chemical composition of EMR (mass fraction, %).

Component	CaO	SiO_2_	MgO	SO_3_	Fe_2_O_3_	TiO_2_	K_2_O	Na_2_O	MnO	CuO
Mass fraction (%)	5.65	29.64	1.89	17.16	3.3	0.38	2.09	0.45	2.56	0.01

**Table 2:** Basic physicochemical characteristics of the raw sewage sludge.

Parameter	Unit	Value
Moisture content	wt.% (wet basis)	85
pH		6.9
Organic matter (as volatile solids)	wt.% (dry basis)	57.32
Ash content	wt.% (dry basis)	42.50

**Table 3:** Major oxide composition of the raw sewage sludge determined by XRF.

Component	SiO_2_	Fe_2_O_3_	Al_2_O_3_	P_2_O_5_	CaO	SO_3_	MgO	K_2_O	TiO_2_	ZnO	MnO	MoO_3_
Mass fraction (%)	29.1	22.26	14.49	11.5	9.1	7.42	2.33	2.01	0.76	0.25	0.17	0.04

**Table 4:** Textural properties of SBC and compressive strengths of the corresponding ECB specimens under different SBC pyrolysis conditions.

	SBC at 400 °C for 90 min	SBC at 500 °C for 90 min	SBC at 600 °C for 90 min	SBC at 500 °C for 60 min	SBC at 500 °C for 120 min
specific surface area (m^2^/g)	49.44	55.06	42.94	38.71	31.64
Average pore size (nm)	11.64	10.52	10.84	11.8	12
Pore volume (cm^3^/g)	0.15	0.14	0.12	0.12	0.1
ECB compressive strength at 7 d (MPa)	1.63	2.19	1.26	1.86	1.66
ECB compressive strength at 14 d (MPa)	1.81	2.32	1.55	1.96	1.71
ECB compressive strength at 28 d (MPa)	2.23	2.44	1.81	2.14	1.73

**Table 5:** Mix proportions of ECB specimens with different SBC dosages.

Mixing Parameter	0% SBC	3% SBC	5% SBC	7% SBC
EMR (%)	85	85	85	85
OPC (%)	15	12	10	8
Water-to-solid ratio (%)	30	30	30	30

**Table 6:** The revised BCR sequential extraction conditions.

Step	Mn Operational	Extractant and Extraction Conditions	Post-Treatment
1	Acid-soluble/exchangeable	1.00 g sample + 40 mL 0.11 mol/L CH_3_COOH; shaken for 16 h at room temperature	Centrifugation; supernatant collected; residue washed
2	Reducible	Residue from Step 1 + 40 mL 0.5 mol/L NH_2_OH·HCl (pH ≈ 1.5); shaken for 16 h at room temperature	Centrifugation; supernatant collected; residue washed
3	Oxidizable	Residue from Step 2 + 10 mL 8.8 mol/L H_2_O_2_; 1 h at room temperature followed by 1 h at 85 ± 2 °C; another 10 mL H_2_O_2_ added and heated at 85 ± 2 °C for 1 h; after cooling, 50 mL 1 mol/L NH_4_OAc (pH 2.0) added and shaken for 16 h	Centrifugation; supernatant collected
4	Residual	The final residue was digested with a mixture of HCl and HNO_3_ (3:1, *v*/*v*) for residual Mn determination.	Residual Mn concentration determined

**Table 7:** Inventory and emission factors used for the carbon-footprint assessment.

Parameter	Value	Source
OPC, 0%SBC	176.47 kg/t EMR	This study
OPC, 5%SBC	117.65 kg/t EMR	This study
SBC	58.82 kg/t EMR	This study
Dry sludge required	94.39 kg/t EMR	This study
OPC EF	0.829 kg CO_2_-eq/kg	Literature
Sludge dewatering + pyrolysis	1.607 kg CO_2_-eq/kg DS	Sun et al. [27]
Coal co-incineration	1.13333 kg CO_2_-eq/kg DS	Yang et al. [28].
Self-sustained incineration	0.35089 kg CO_2_-eq/kg DS	Yang et al. [28].

## Data Availability

The original contributions presented in this study are included in the article. Further inquiries can be directed to the corresponding author.
